# Supplementary material for: Sex- and context-dependent effects of acute isolation on vocal and non-vocal social behaviors in mice
Source: PLoS One. 2021 Sep 1;16(9):e0255640. doi: 10.1371/journal.pone.0255640 (PMC8409668; doi:10.1371/journal.pone.0255640)
Supplement: S1 File — (PDF) [file pone.0255640.s001.pdf]

S1 Fig. Effects of acute isolation on latency to first USV in same-sex and opposite-sex interactions

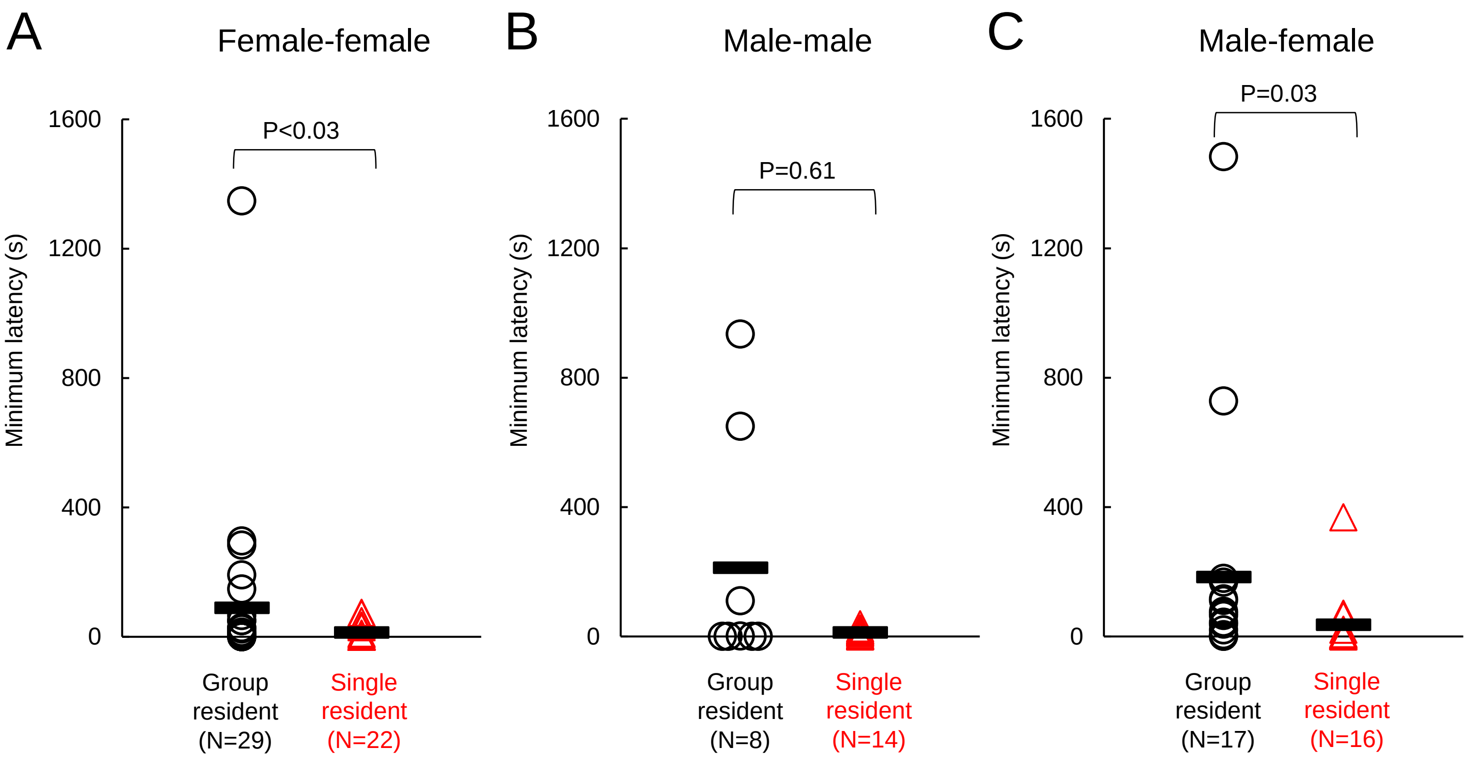

S1 Fig. Effects of acute isolation on latency to first USV in same-sex and opposite-sex interactions. Latency to the first recorded USV is shown for female-female social encounters (A), male-male encounters (B), and male-female encounters (C). Trials with 0 USVs are excluded.

S2 Fig (1/3). Female-female ethograms, group-housed residents

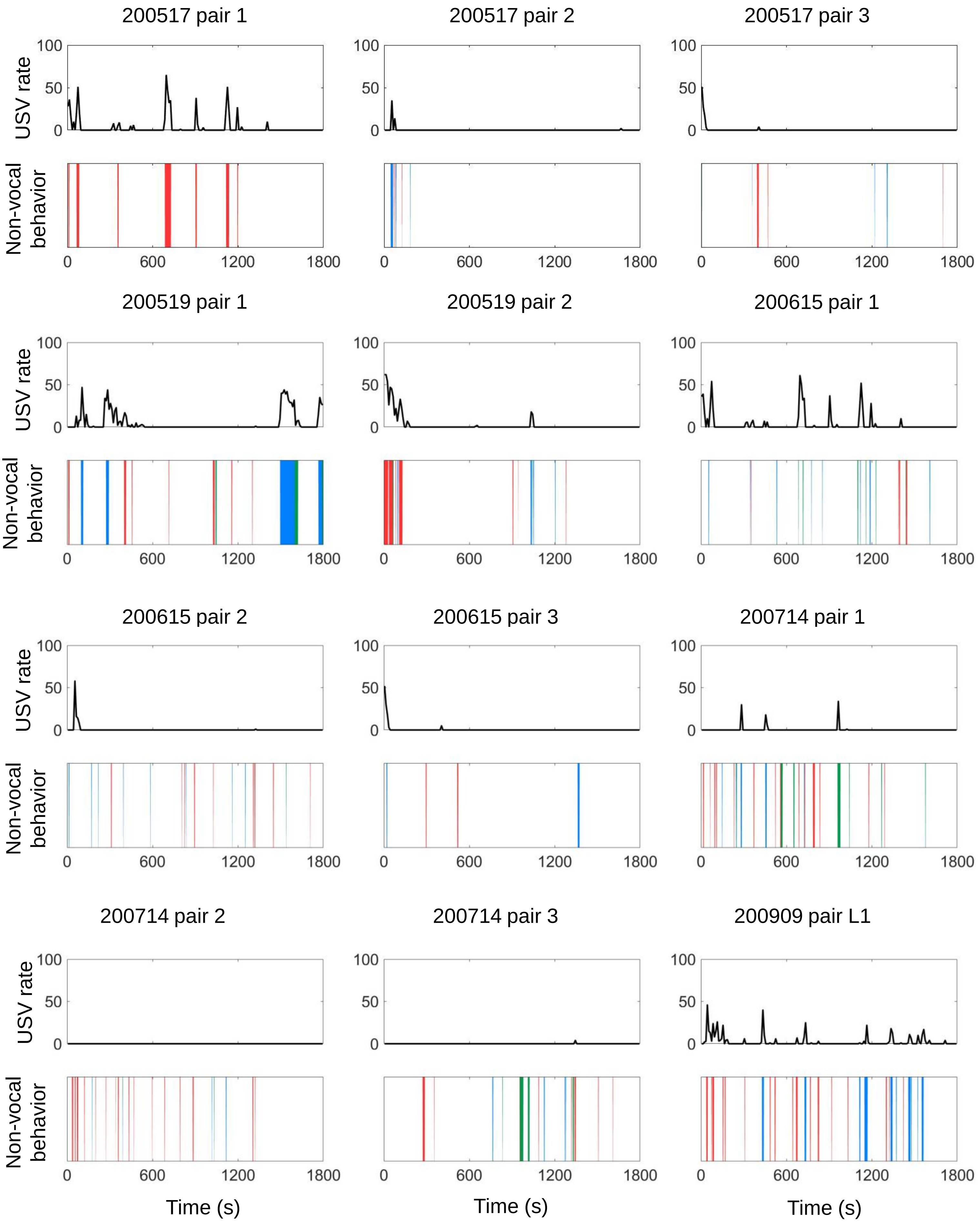

S2 Fig (2/3). Female-female ethograms, group-housed residents

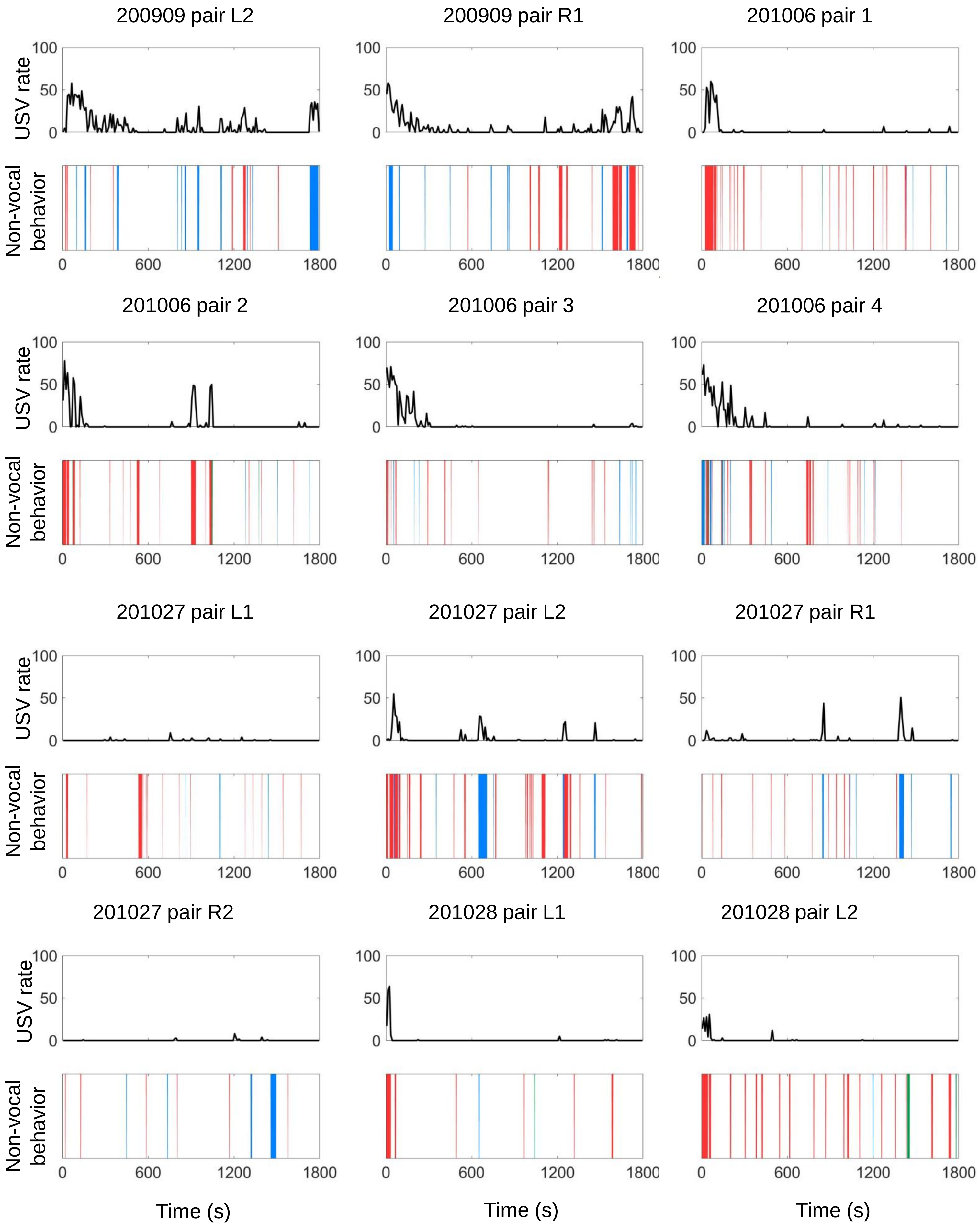

S2 Fig (3/3). Female-female ethograms, group-housed residents

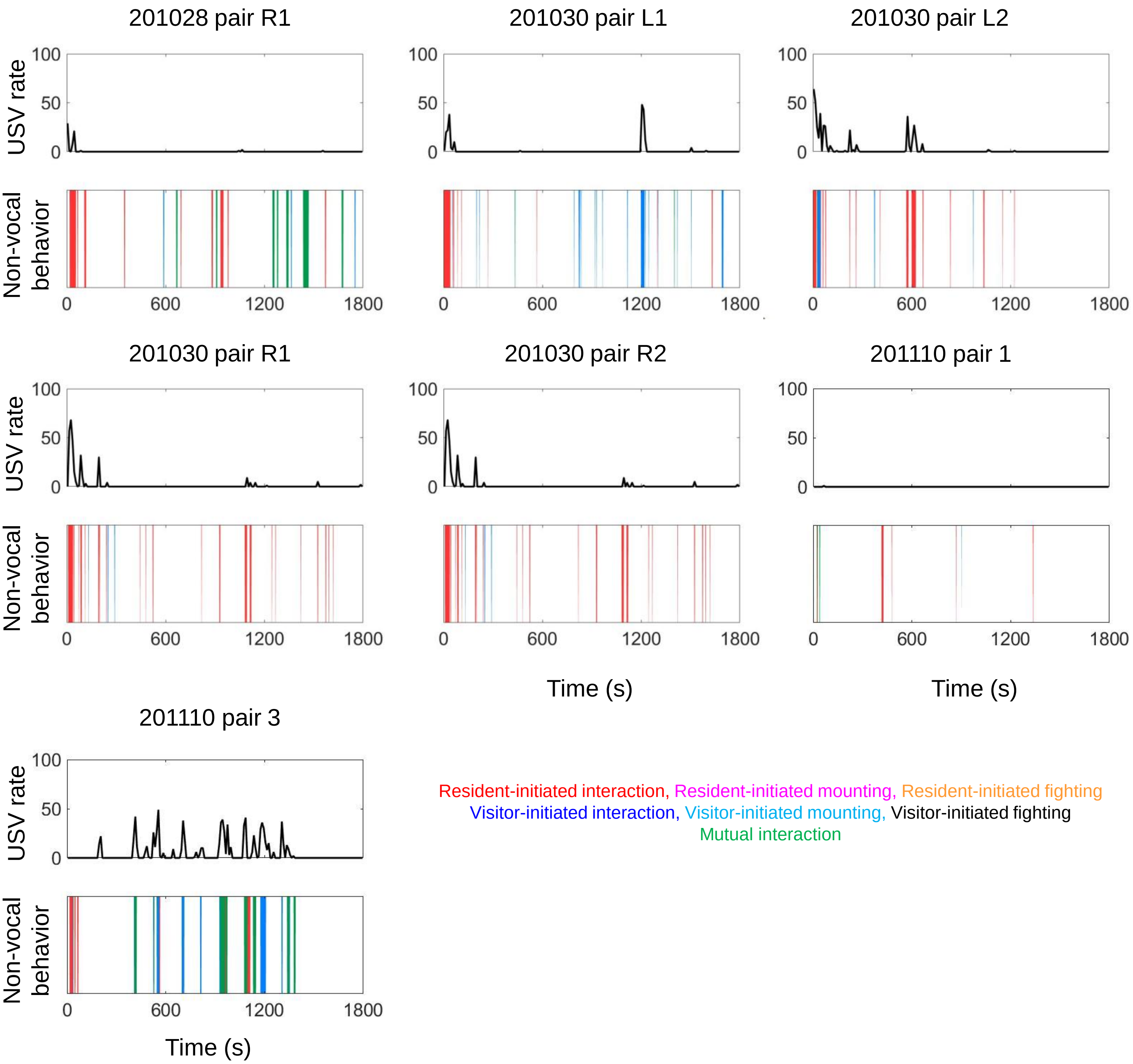

S2 Fig. Female-female ethograms, group-housed residents. Ethograms are shown for each female-female trial with a group-housed resident. The top half of each plot shows USV rate over time (total USVs in each 10s-long bin), and the bottom half of each plot shows the occurrence of different non-vocal social behaviors over time. Red, resident-initiated interaction; magenta, resident-initiated mounting; orange, resident-initiated fighting; blue, visitor-initiated interaction; cyan, visitor-initiated mounting; black, visitor-initiated fighting; green, mutual interaction; white, not interacting.

S3 Fig (1/2). Female-female ethograms, single-housed resident

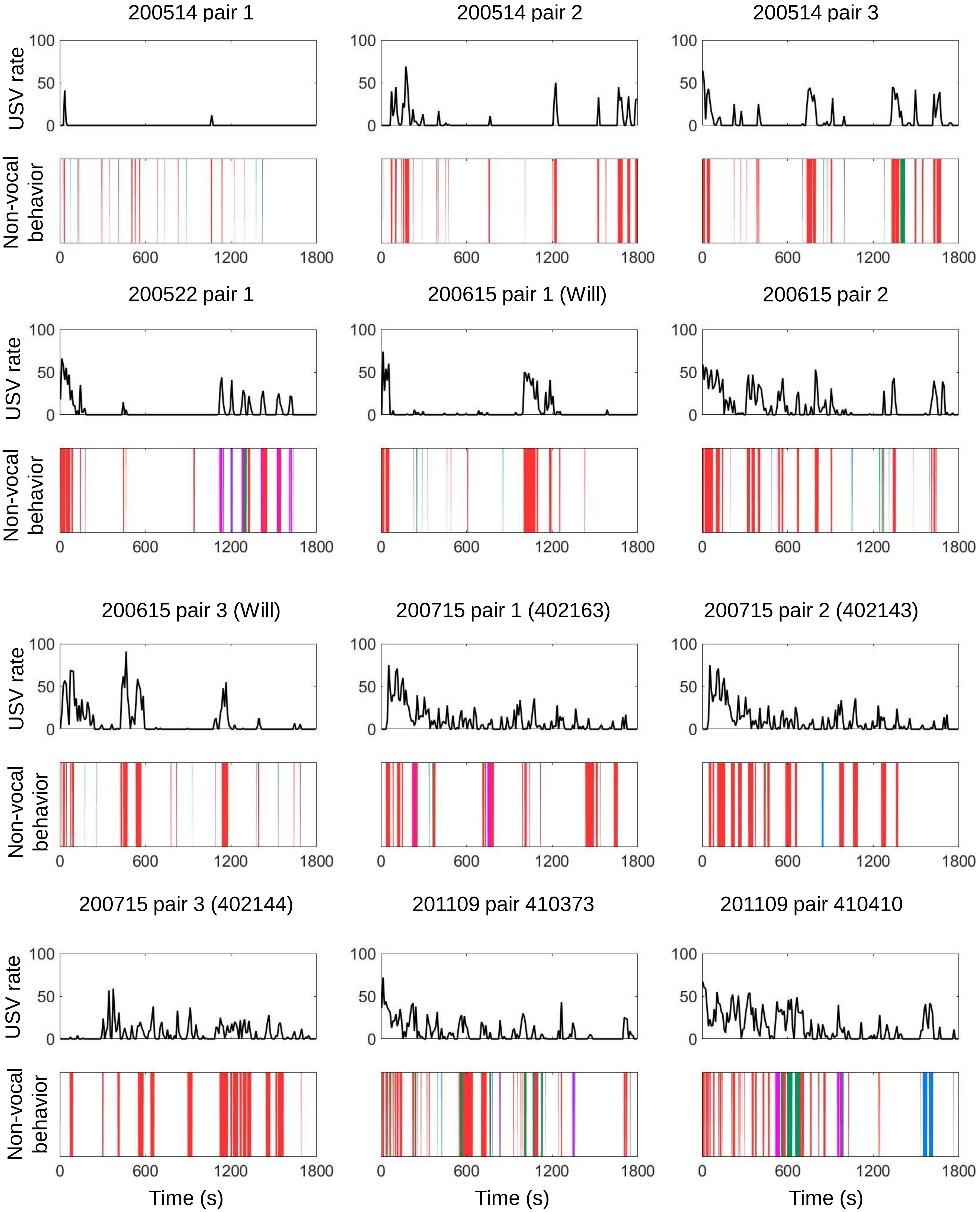

S3 Fig (2/2). Female-female ethograms, single-housed resident

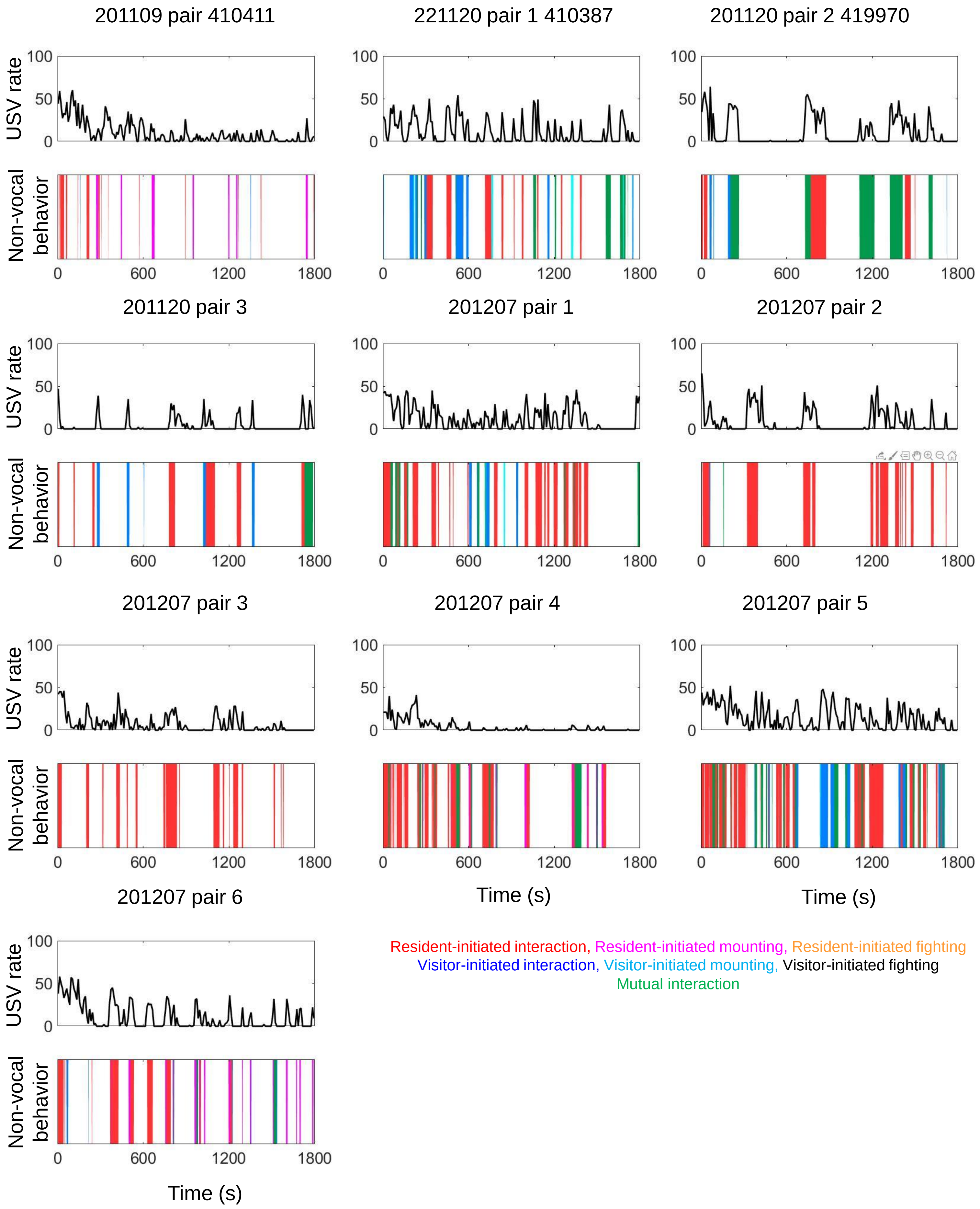

S3 Fig. Female-female ethograms, single-housed residents. Ethograms are shown for each female-female trial with a single-housed resident. The top half of each plot shows USV rate over time (total USVs in each 10s-long bin), and the bottom half of each plot shows the occurrence of different non-vocal social behaviors over time. Red, resident-initiated interaction; magenta, resident-initiated mounting; orange, resident-initiated fighting; blue, visitor-initiated interaction; cyan, visitor-initiated mounting; black, visitor-initiated fighting; green, mutual interaction; white, not interacting.

S4 Fig (1/2). Male-male ethograms, group-housed resident

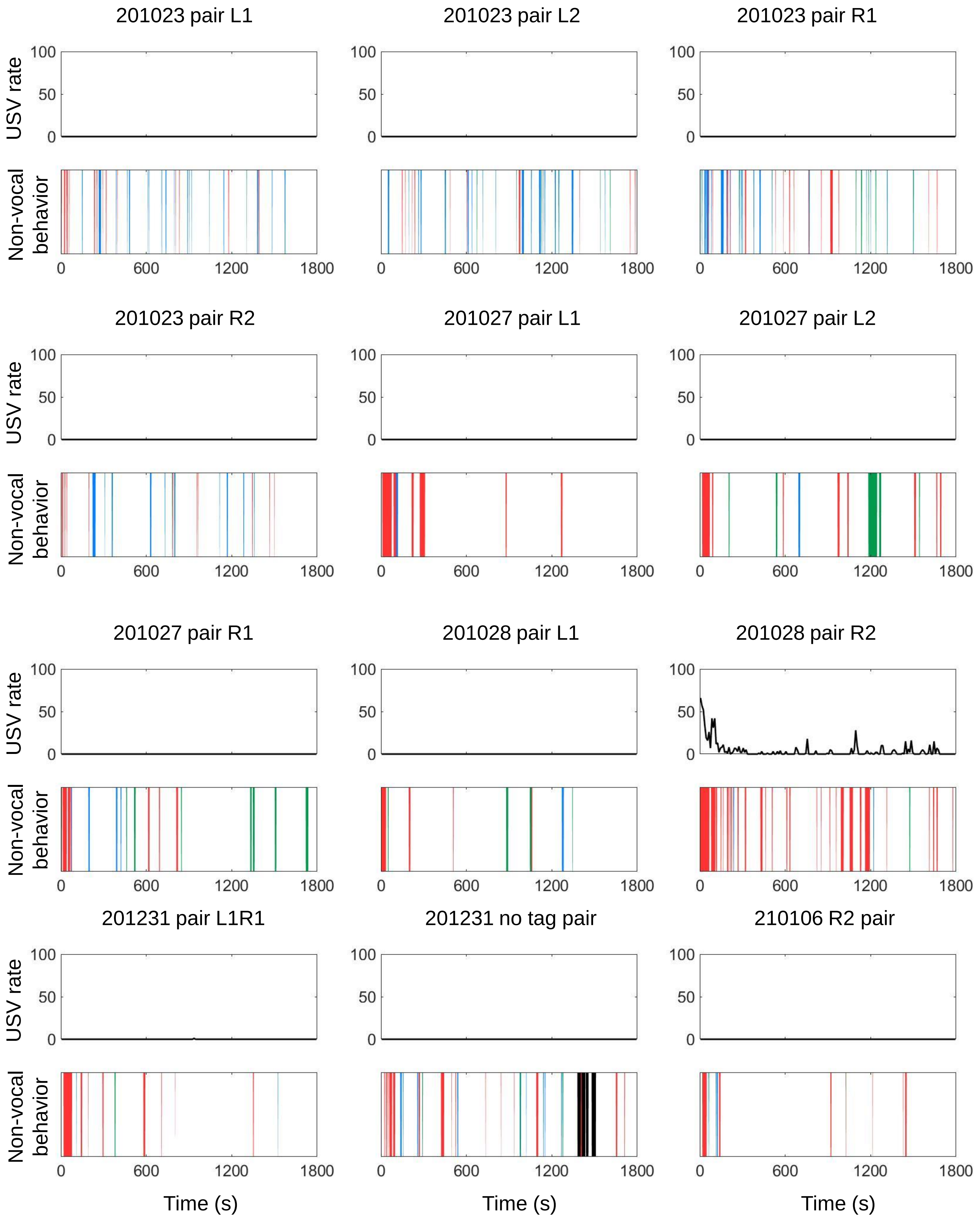

S4 Fig (2/2). Male-male ethograms, group-housed resident

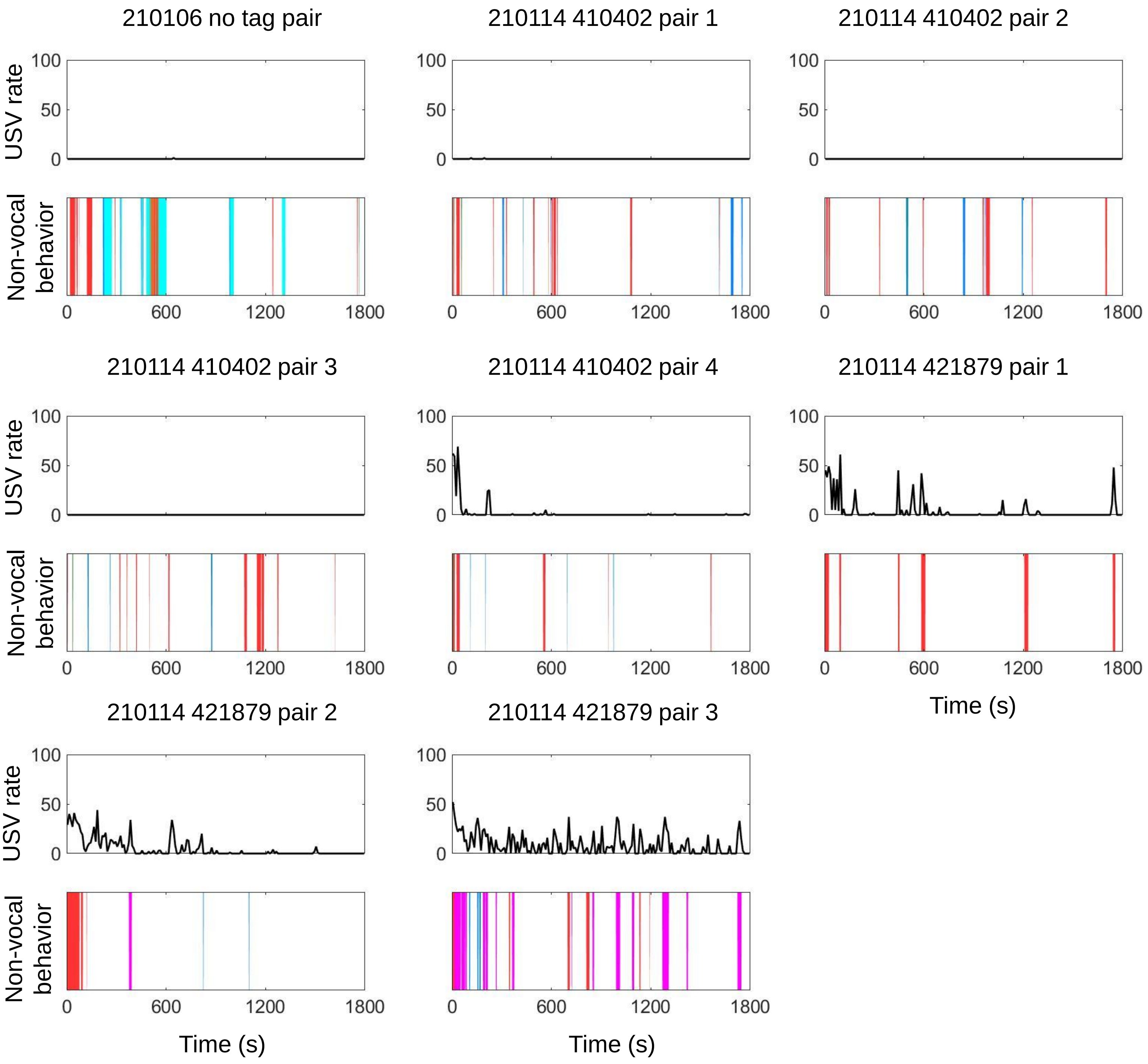

Resident-initiated interaction, Resident-initiated mounting, Resident-initiated fighting
Intruder-initiated interaction, Intruder-initiated mounting, Intruder-initiated fighting
Mutual interaction

S4 Fig. Male-male ethograms, group-housed residents. Ethograms are shown for each male-male trial with a group-housed resident. The top half of each plot shows USV rate over time (total USVs in each 10s-long bin), and the bottom half of each plot shows the occurrence of different non-vocal social behaviors over time. Red, resident-initiated interaction; magenta, resident-initiated mounting; orange, resident-initiated fighting; blue, visitor-initiated interaction; cyan, visitor-initiated mounting; black, visitor-initiated fighting; green, mutual interaction; white, not interacting.

S5 Fig. (1/2). Male-male ethograms, single-housed resident

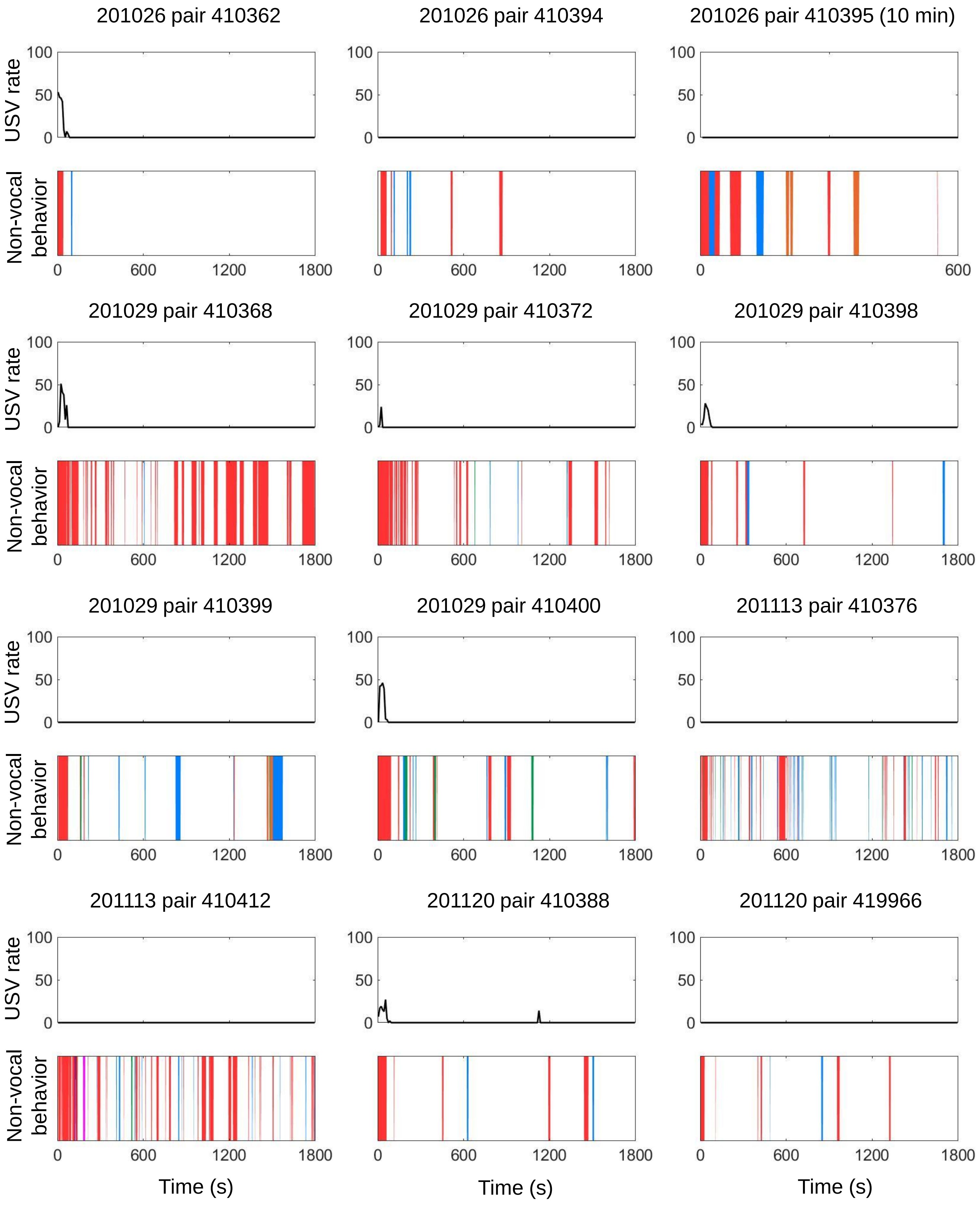

S5 Fig (2/2). Male-male ethograms, single-housed resident

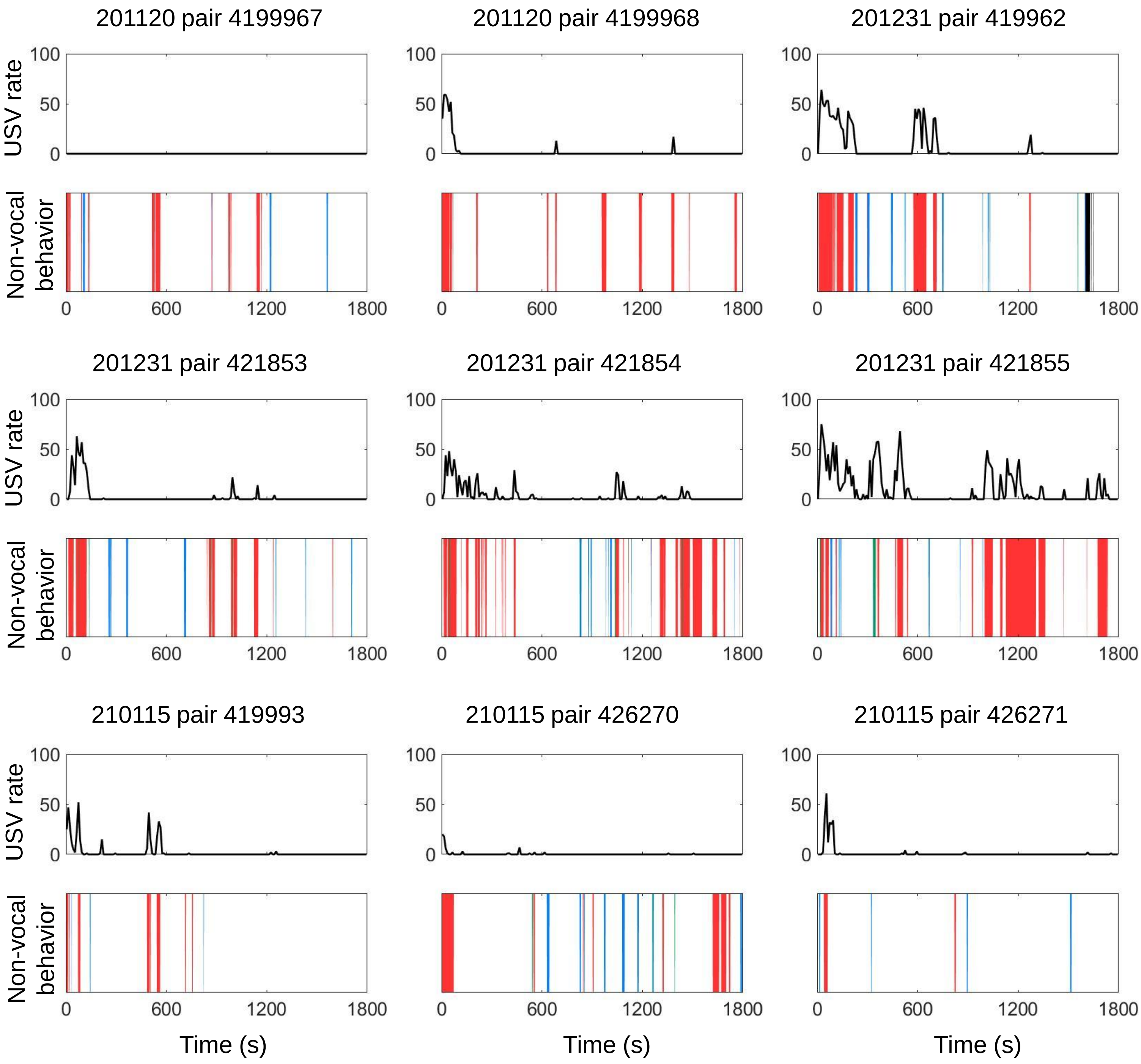

Resident-initiated interaction, Resident-initiated mounting, Resident-initiated fighting
Intruder-initiated interaction, Intruder-initiated mounting, Intruder-initiated fighting
Mutual interaction

S5 Fig. Male-male ethograms, single-housed residents. Ethograms are shown for each male-male trial with a single-housed resident. The top half of each plot shows USV rate over time (total USVs in each 10s-long bin), and the bottom half of each plot shows the occurrence of different non-vocal social behaviors over time. Red, resident-initiated interaction; magenta, resident-initiated mounting; orange, resident-initiated fighting; blue, visitor-initiated interaction; cyan, visitor-initiated mounting; black, visitor-initiated fighting; green, mutual interaction; white, not interacting.

S6 Fig (1/2). Male-female ethograms, group-housed resident

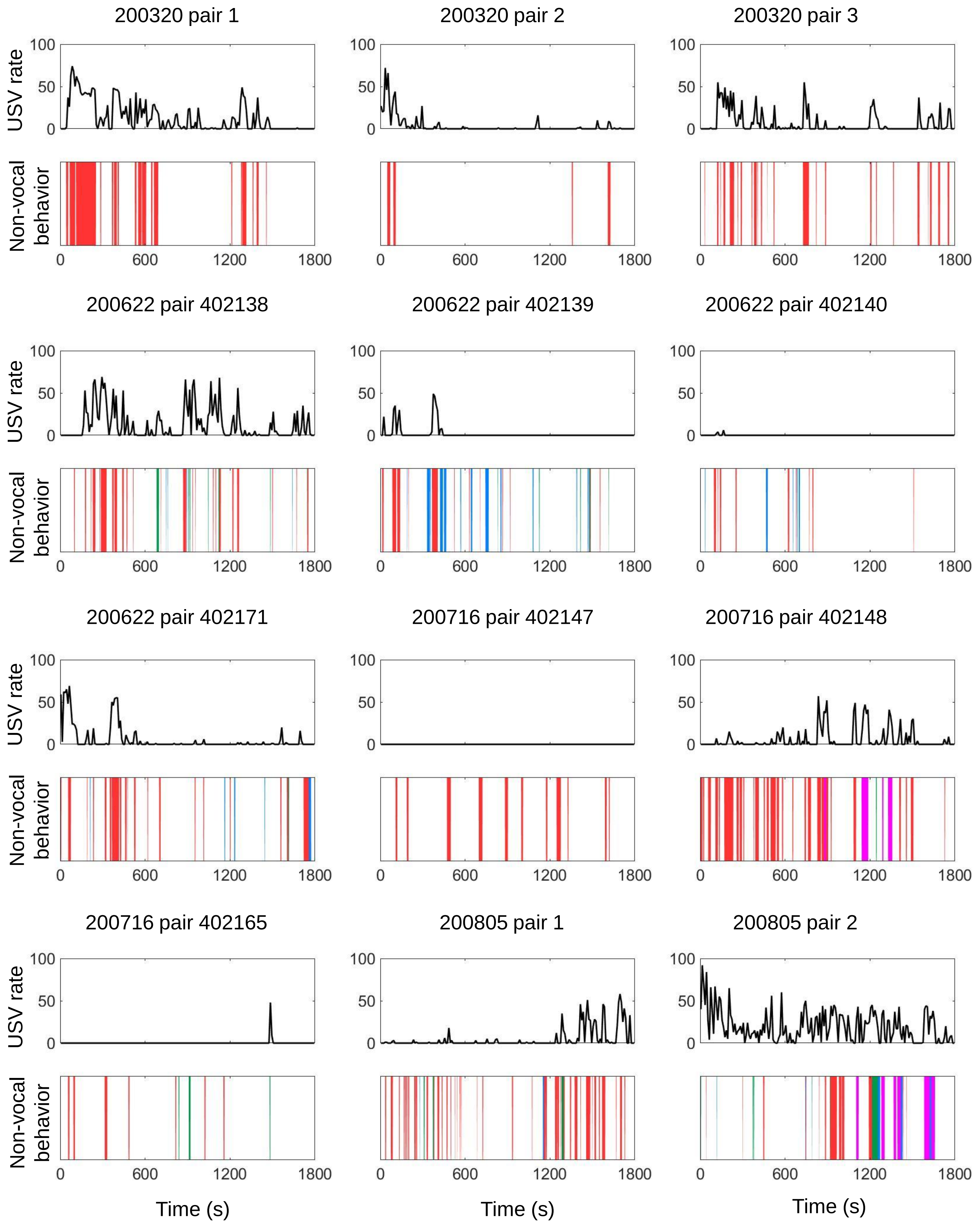

S6 Fig (2/2). Male-female ethograms, group-housed resident

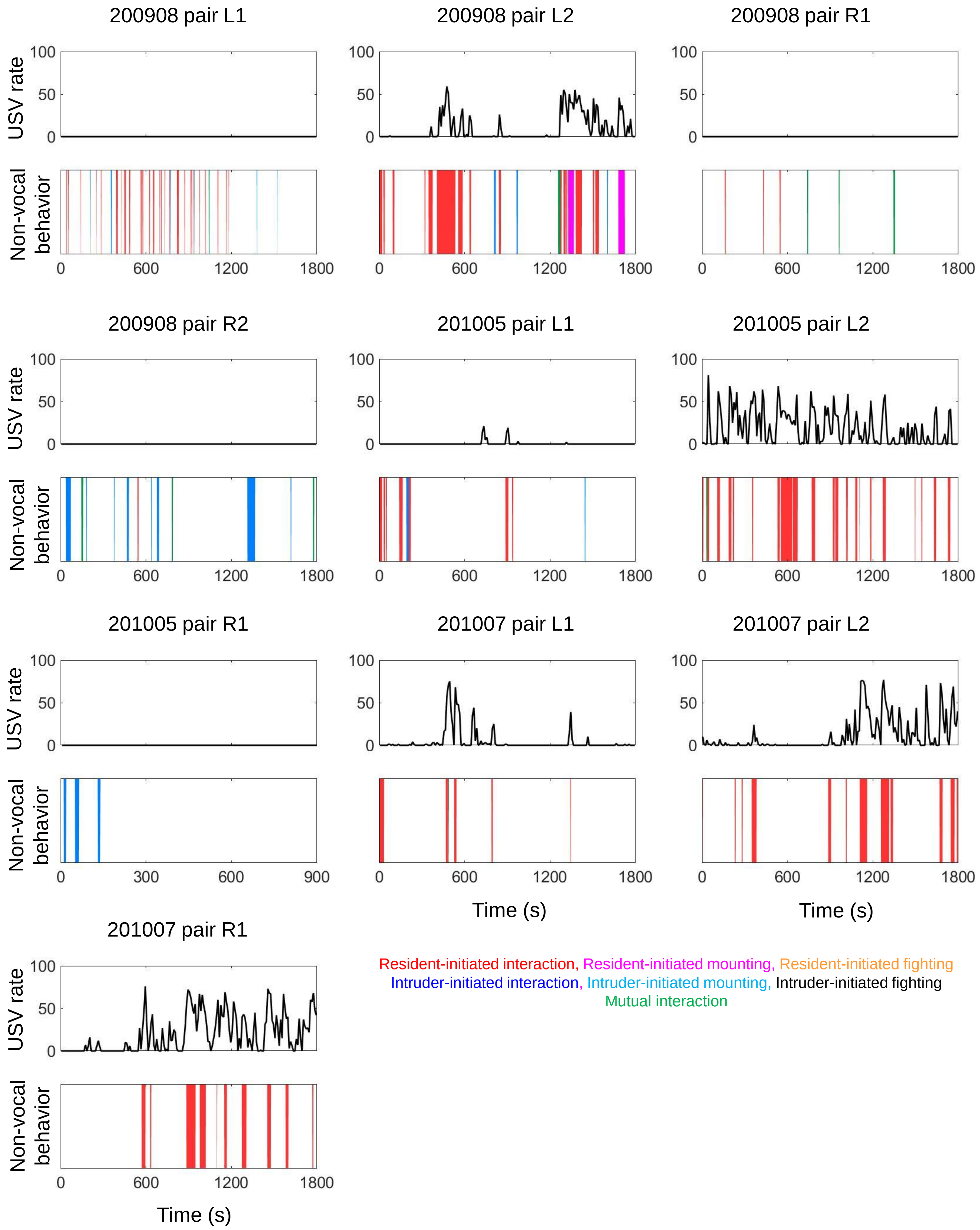

S6 Fig. Male-female ethograms, group-housed residents. Ethograms are shown for each male-female trial with a group-housed resident. The top half of each plot shows USV rate over time (total USVs in each 10s-long bin), and the bottom half of each plot shows the occurrence of different non-vocal social behaviors over time. Red, resident-initiated interaction; magenta, resident-initiated mounting; orange, resident-initiated fighting; blue, visitor-initiated interaction; cyan, visitor-initiated mounting; black, visitor-initiated fighting; green, mutual interaction; white, not interacting.

S7 Fig (1/2). Male-female ethograms, single-housed resident

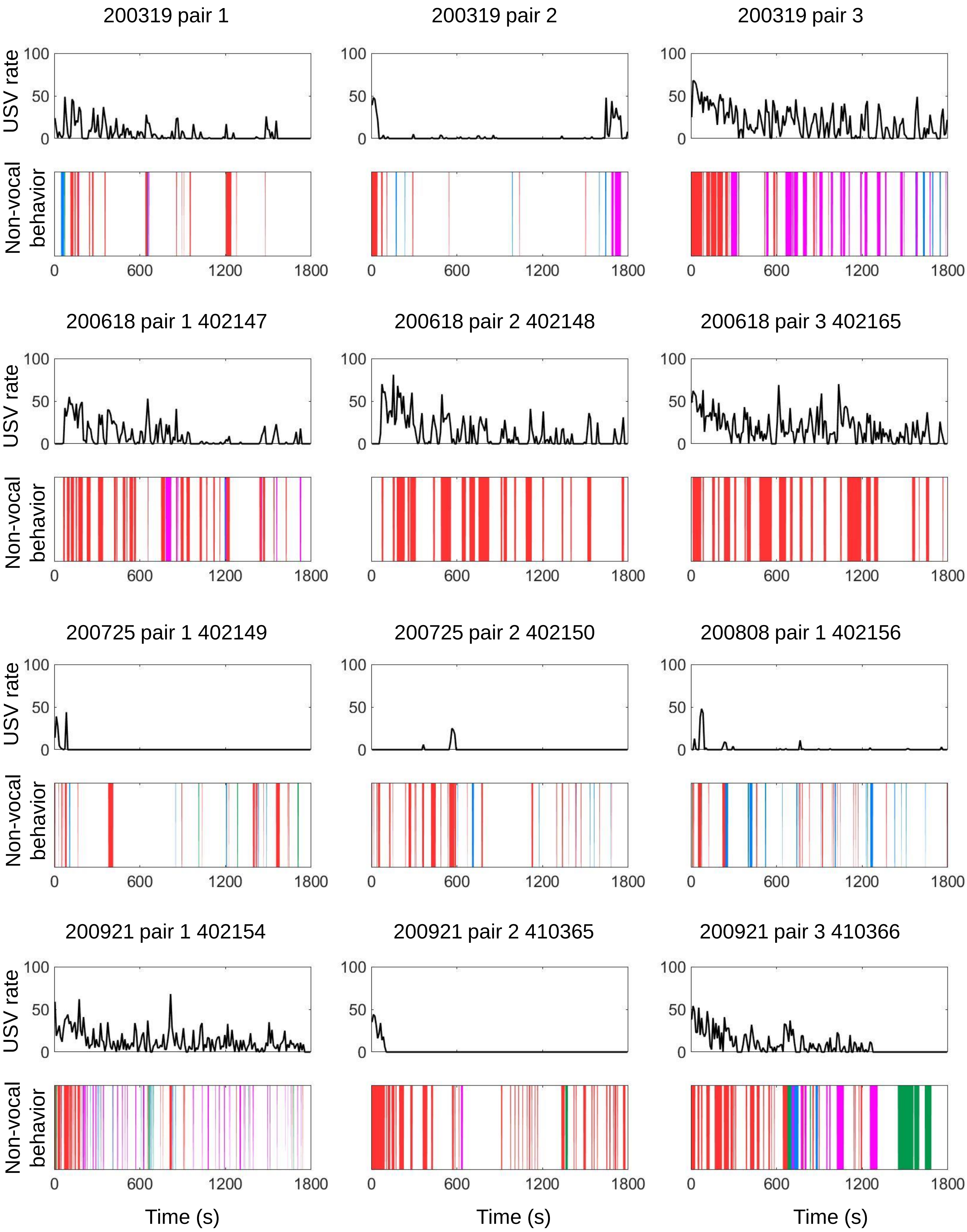

S7 Fig (2/2). Male-female ethograms, single-housed resident

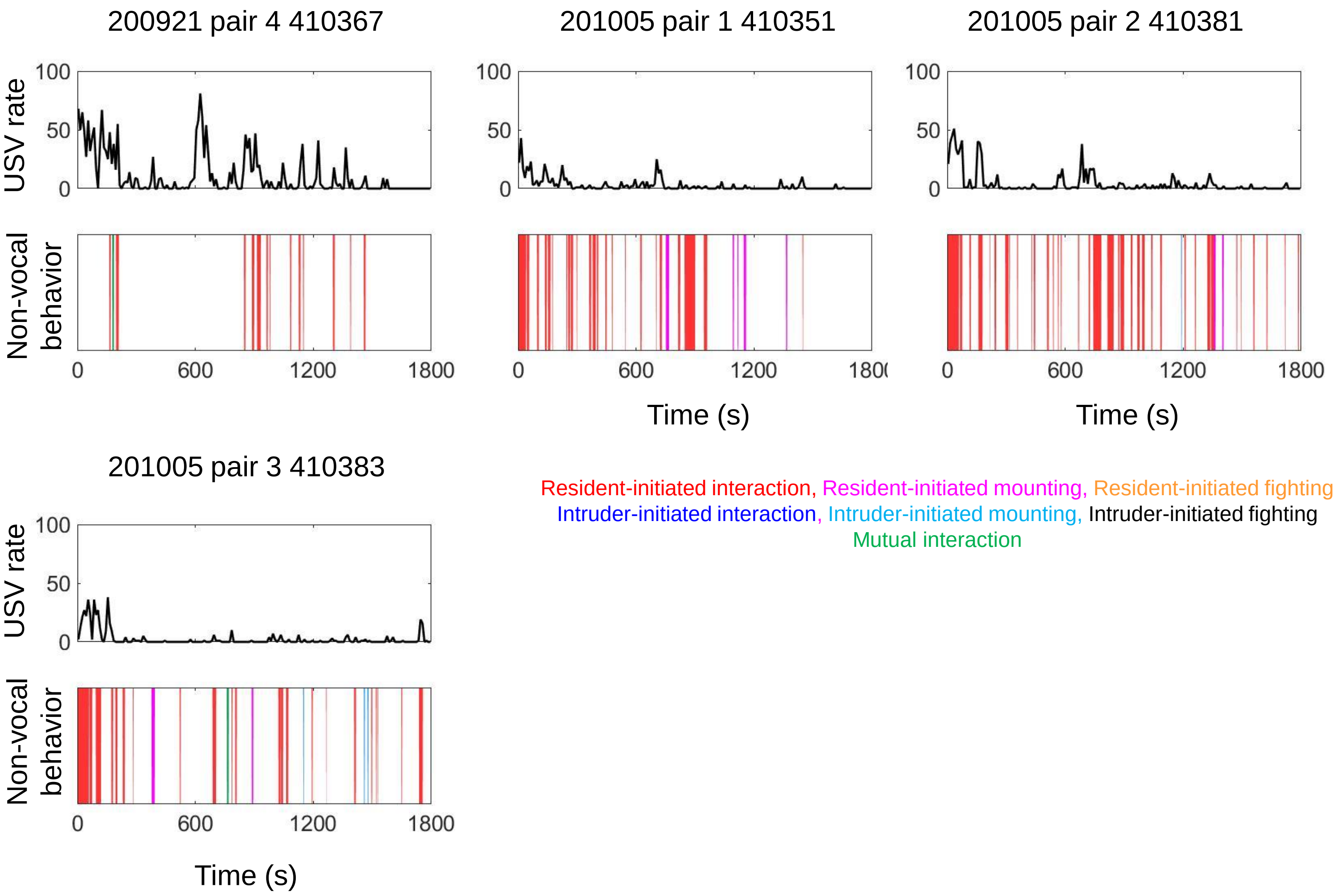

S7 Fig. Male-female ethograms, single-housed residents. Ethograms are shown for each male-female trial with a single-housed resident. The top half of each plot shows USV rate over time (total USVs in each 10s-long bin), and the bottom half of each plot shows the occurrence of different non-vocal social behaviors over time. Red, resident-initiated interaction; magenta, resident-initiated mounting; orange, resident-initiated fighting; blue, visitor-initiated interaction; cyan, visitor-initiated mounting; black, visitor-initiated fighting; green, mutual interaction; white, not interacting.

S1 Table. Statistical Summary

Figure Number	Statistical Test	P value	Description
Fig 1A, left	Mann Whitney	<0.0001	USV counts, female-female pairs
Fig 1B, left	Mann Whitney	0.15	USV counts, male-male pairs
Fig 1C, left	Mann Whitney	0.44	USV counts, male-female pairs
Fig S1A	Mann Whitney	0.03	USV latency, female-female pairs
Fig S1B	Mann Whitney	0.61	USV latency, male-male pairs
Fig S1C	Mann Whitney	0.03	USV latency, male-female pairs
text only	z-test for 2 independent proportions	0.02	proportion of male-male trials with >25 USVs
Fig 2A, left	Mann Whitney	<0.001	time spent interacting, female-female pairs
Fig 2A, middle	Mann Whitney	0.08	time spent interacting, male-male pairs
Fig 2A, right	Mann Whitney	0.08	time spent interacting, male-female pairs
Fig. 2B	two-way ANOVA, repeated measures on one factor<br>(A, B, and interaction)	<0.0001<0.0001<0.0001	differences in female-female non-vocal social behaviors
		<0.0001<0.0001<0.0001	differences in male-male non-vocal social behaviors
		>0.05<br><0.001<br>>0.05	differences in male-female non-vocal social behaviors

Fig. 2B	Post-hoc tests (resident-initiated, visitor-initiated, mutual)	<0.0001 0.18. 0.06	differences in female-female non-vocal social behaviors
		0.003 0.25 0.26	differences in male-male non-vocal social behaviors
text and Fig. 2C	z-test for 2 independent proportions	p<0.001	proportion of female-female trials with mounting
		p=0.27	proportion of male-male trials with mounting
		p=0.005	proportion of male-female trials with mounting
text only	z-test for 2 independent proportions	0.41	proportion of male-male trials with fights
Fig. 3A, left	linear regression	0.002<br>0.001	Female-female, group-housed resident<br>Female-female, single-housed resident
Fig. 3A, middle	linear regression	0.83<br>0.02	Male-male, group-housed resident<br>Male-male, single-housed resident
Fig. 3A, right	linear regression	0.003<br>0.006	Male-female, group-housed resident<br>Male-female, single-housed resident
Fig. 3B	two-way ANOVA, repeated measures on one factor (A, B, and interaction)	<0.001 <0.001 0.004	differences in proportion female-female USVs produced during different non-vocal social behaviors
		<0.001 <0.001 <0.001	differences in proportion male-male USVs produced during different non-vocal social behaviors
		>0.05 <0.001 >0.05	Differences in proportion male-female USVs

			produced during different non-vocal social behaviors
Fig. 3B	Post-hoc tests (resident-initiated, visitor-initiated, mutual)	0.034 0.12 0.05	differences in proportion female-female USVs produced during different non-vocal social behaviors
		0.006 0.96<br>0.96	differences in proportion male-male USVs produced during different non-vocal social behaviors
