# Supplementary material for: Sex- and context-dependent effects of acute isolation on vocal and non-vocal social behaviors in mice
Source: PLoS One. 2021 Sep 1;16(9):e0255640. doi: 10.1371/journal.pone.0255640 (PMC8409668; doi:10.1371/journal.pone.0255640)
Supplement: Attachment — Submitted filename: PONE D 21 12891 review 5.12.21.pdf [file pone.0255640.s002.pdf]

The manuscript, Sex and context-dependent effects of acute isolation on vocal and non-vocal social behaviors in mice, presents novel data on how mice react to three days of social isolation. The work is potentially important to understanding the relationship between social motivation and ultrasonic vocalizations (USV's) and thus advances understanding of the function of these signals, but the context of communication is not strongly presented in the introduction or discussion. I also have some questions about the scientific rationale and the experimental methods that are critical to the interpretation of the findings. While some of my questions are critical to evaluating the interpretation offered by the authors, I offer my suggestions to strengthen the paper. These are novel data and placed in the correct context could help advance understanding of USV's and social motivation in mice. I listed my broad concerns and specific questions/suggestions by section, below.

Comments on the Introduction:
I find it surprising that the paper begins with a review of human behavior rather than mouse behavior. I do understand that lab mice are used as a model for human health but evaluating the strength of the scientific rationale for examining the effects of social isolation in mice requires a reader understand of mouse behavior. Later in the discussion some information is provided about mouse social interactions but I would find the addition of a paragraph in the introduction justifying how the study is relevant to understanding mice very compelling. This paragraph should also review what is known about USV's to clarify how this work advances understanding of these signals.

Line 30- A review of the frequency of social encounters between unfamiliar individuals in the wild would justify how the data collected explain mouse behavior. While mice are used as a model for humans, the data collected here do not inform human health. Thus, the paper opens with one context and closes with a conclusion completely unrelated to this context, undermining the significance of the results.

Line 38-43 – The gap identified in the literature is clear but the importance of filling the gap is not. Why is it important to understand acute effects of isolation across contexts? Again, more information in the introduction about sex differences in social behavior of mice would justify these comparisons and explain why this work is important.

Line 53 – The justification for this work in this version of the manuscript seems to be that this set of studies have not been done before. The argument will be stronger if you explain how this work will advance understanding – what the study contributes to the field.

Comments on the Methods:
If possible, I recommend explaining the methods before the results because the interpretation of the results depends upon understanding the study design. Moreover, the methods as they are currently written left me with important questions that make interpreting the findings impossible. First, it is not completely clear if different subjects were used in each treatment of if this was a repeated measures design (e.g., line 65, 78, 394), which could lead to very different conclusions. Second, it is not clear if each focal animal was unfamiliar with its visitor as not all

mice were naïve. These point are critical to ruling out the possibility that behavioral differences reflect social memory of companions. Making these points explicit, early in the paper will keep you reader 'on your team' for the interpretation.

Line 374 – How many subjects of each sex were focal subjects? Was each focal subject tested in only one social context or multiple contexts? Without this information it is impossible to determine if findings reflect actual treatments or if they could be influenced by prior experiences in previous trials. I would make this clear in the last paragraph of the introduction and also very specific here in the methods.

Line 376 -382 – It is unclear if all subjects were moved from their home cage to a recording chamber or if group housed subjects were kept in their home cage and only the sibling removed. This is important because if focal subjects from the treatments were handled differently then the potential impacts of this experience need to be addressed in the interpretation of the data.

Line 410 – The section on statistics needs to be fully developed as a mix of parametric and non-parametric tests are used and the rationale for choosing specific tests is not clear.

Comments on Results
The results in the present draft contain some methodological details and interpretation that would be more effective in the methods and discussion, respectively.

Line 78 – What is meant by 'we first measured'? Were trails not presented in balanced order?

Line 100 - Switching methods to compare proportions instead of behavioral counts or latencies reads as though it is data mining. Again, presenting the statistics, justification for the statistical approaches used, and applying those approaches evenly to all treatments will address this concern.

Line 111 – Are you citing your first finding here? Was the second study not conceived of until after the first was completed?

Comments on the Discussion

To reiterate the concern presented with the methods section, the interpretation of the data in the discussion is interesting but cannot be evaluated until clear methods and experimental design are provided. If animals were not unfamiliar with one another and/or if they had experienced prior trials then the differences in social context may reflect prior experience, not sex-differences. Additionally, the strength of these data are the contribution to understanding the function of USV's yet very little is discussed about the theoretical framework of communication and how these data add to our understanding.

Line 265 – Are female mice in nesting groups often genetically related as occurs in many other mammals? Do you have information about the genetic relatedness of subjects in these tests?

Line 301 – Might the isolated subject and visitor have different motivations? What reasons might a resident be more aggressive and a visitor more affiliative? The discussion later about sex differences in social interactions is helpful but are there also studies on territoriality/home range that might be important for context, here?

Line 330 – There are several theoretical ideas about animal communication that these data could be relevant to and it is a missed opportunity not to address this contribution of the data. Communication signals are argued to be emotive, manipulative, or referential. The data presented here support the interpretation that USV's are emotive and reflect the internal state of an animal, thus advancing understanding of these signals. A quick review of a book on animal communication such as Nowicki and Searcy's *Evolution of Animal Communication* could help frame this context.

Line 358 – I do not understand what 'vice versa' refers to, here.
